# Baseline Body Composition Characteristics and Overall Survival in Young Women with Breast Cancer: Matched Case–Control Study Nested Within a Cohort

**DOI:** 10.3390/tomography12040054

**Published:** 2026-04-08

**Authors:** Aynur Aktas, Diptasree Mukherjee, Danielle Boselli, Brandon N. VanderVeen, Lejla Hadzikadic-Gusic, Rebecca S. Greiner, Michelle L. Wallander, Declan Walsh, Kunal C. Kadakia

**Affiliations:** 1 Department of Supportive Oncology, Atrium Health Levine Cancer, Charlotte, NC 28204, USA; dr.diptasree@gmail.com (D.M.); rebecca.greiner@advocatehealth.org (R.S.G.); declan.walsh@advocatehealth.org (D.W.); kunal.kadakia@advocatehealth.org (K.C.K.); 2 Atrium Health Wake Forest Baptist Comprehensive Cancer Center, Winston-Salem, NC 27157, USA; lejla.hadzikadic-gusic@advocatehealth.org (L.H.-G.); michelle.wallander@advocatehealth.org (M.L.W.); 3 Department of Biostatistics and Data Sciences, Atrium Health Levine Cancer, Charlotte, NC 28204, USA; danielle.boselli@advocatehealth.org; 4 Department of Cancer Biology, School of Medicine, Wake Forest University, Winston-Salem, NC 27157, USA; brandon.vanderveen@advocatehealth.org; 5 Department of Surgery, Division of Surgical Oncology, Atrium Health Levine Cancer, Charlotte, NC 28204, USA; 6 Department of Solid Tumor Oncology, Atrium Health Levine Cancer, Charlotte, NC 28204, USA

**Keywords:** body composition, breast neoplasms, mortality, neoplasm, prognosis, computed tomography

## Abstract

Background/Objectives: Young women with breast cancer (aged ≤ 40 years) have distinct prognostic characteristics, yet little is known about how modifiable body composition factors influence outcomes in this age group. This study examined whether CT-derived body composition measures could identify thresholds that predict overall survival (OS). Methods: This was a single-center, 10-year, matched case–control study nested within a cohort, utilizing retrospectively collected data. Using an institutional database (2009–2018) and the initial cohort of 112 patients, we performed a subset analysis of patients with stage I–III breast cancer at diagnosis who had available pretreatment CT scans to estimate associations with body composition metrics and OS. The final analytic dataset included 89 individuals (49 survivors and 40 deceased). CT scans at the L3 level were analyzed using Slice-O-Matic software to quantify visceral (VAT), subcutaneous (SAT), intermuscular (IMAT), total adipose tissue (TAT), skeletal muscle density (SMD), skeletal muscle gauge (SMG), and skeletal muscle index (SMI). Cox proportional hazard models determined optimal cutpoints for OS. Multivariable models included adjustments for disease stage and hormone receptor status. Results: The median age was 35 (IQR, 32–38); 47% were White and 37% were Black. The majority (78%) were not Hispanic or Latina. Most (67%) were overweight/obese. Specific thresholds for IMAT index (>2.57), VAT (>31.38), and SMG (<2419.89) were associated with worse survival (all *p* < 0.05), while no cutpoints were identified for other variables. Conclusions: These findings show that muscle fat infiltration and reduced muscle quality have important prognostic value in young women with breast cancer. Exploratory cutpoints derived from routine staging CT scans may help inform risk stratification and generate hypotheses for targeted nutritional or exercise interventions, but require validation in larger, independent cohorts before clinical application.

## Simple Summary

Young women with breast cancer often have aggressive disease, and little is known about how body fat and muscle quality affect their survival. In this study, routine CT scans at diagnosis were used to measure different types of body fat and muscle in women aged 40 or younger. Higher fat stored within and around muscles and lower muscle quality were linked to worse long-term survival, whereas simple measures such as body mass index were not. These findings suggest that CT-based body composition may help radiologists and oncologists better assess risk and design future lifestyle or exercise interventions.

## 1. Introduction

Breast cancer remains the most common cancer in women in the United States, accounting for approximately 30% (or 1 in 3) of all new female cancer diagnoses each year [1]. In 2025, an estimated 316,950 new cases of invasive breast cancer were projected to occur among women. Notably, the incidence rate among women under 50 has been increasing at a slightly faster pace (1.4% annually) compared with older age groups [1].

Although breast cancer in younger women is less common, it often presents unique challenges. These include more aggressive tumor biology, advanced disease at presentation, higher rates of local recurrence, and poorer prognosis compared with older patients [2,3,4,5,6]. Furthermore, young women face distinct psychosocial and fertility-related concerns, which further underscores the need for targeted research and intervention strategies [7].

While advances in treatment have improved overall survival (OS) across all groups, young women continue to experience outcome disparities. Increasing attention has focused on modifiable risk factors, particularly those related to body composition, that might be leveraged to improve prognosis in this population [8,9,10,11].

Computed tomography (CT) imaging offers a non-invasive and precise method to quantify body composition metrics such as skeletal muscle index (SMI), visceral adipose tissue (VAT), and intermuscular adipose tissue (IMAT). These CT-derived measures have been associated with survival outcomes across multiple cancer types, including breast cancer, largely in older or mixed-age populations [12,13]. However, data specific to young women with breast cancer remain limited. This represents a significant gap in the literature, given their unique profiles and the potential for early intervention through lifestyle modification.

In a large cohort of 3241 women with non-metastatic breast cancer, sarcopenia (low SMI) was common (34%) and independently predicted higher mortality (HR 1.41) [14]. High total adipose tissue (TAT) also increased risk, whereas BMI did not. Prognosis was poorest when sarcopenia co-occurred with high adiposity. Muscle radiodensity (a surrogate of muscle quality) was not consistently prognostic in that setting, suggesting potential disease stage- or population-specific differences in the relevance of muscle quality measures. Smaller early-stage breast cancer studies have further clarified which CT-derived metrics are clinically informative. Deluche et al. [15] showed that sarcopenia and higher IMAT independently predicted shorter disease-free survival (DFS) and overall survival (OS), while BMI was not prognostic. A higher VAT/SAT ratio was associated with worse outcomes in univariate analyses but did not remain significant after adjustment. Oliveira Júnior et al. [16] found that low skeletal muscle gauge (SMG) and a low VAT/SAT ratio independently predicted shorter DFS, emphasizing that fat distribution offers prognostic information beyond BMI. In a U.S.-based cohort of women ≤ 40 years with stage I–III breast cancer, Aktas et al. [11] showed that higher IMAT and VAT at diagnosis were associated with worse OS, and they proposed exploratory thresholds to identify higher-risk patients. In contrast, when the analysis was restricted to patients with stage IV disease, the association between IMAT and overall survival was not statistically significant [17].

Given these considerations, the present study aims to evaluate CT-derived body composition metrics in young women with breast cancer and identify exploratory threshold values that may be associated with OS. By delineating specific body composition parameters associated with prognosis, this hypothesis-generating research seeks to add data to the endeavors of risk stratification and personalized management strategies for this high-risk group.

## 2. Materials and Methods

### 2.1. Study Design and Study Population

This was a single-center, 10-year, matched case–control study nested within a cohort, utilizing retrospectively collected data. The study protocol received approval from the Wake Forest University Health Sciences Institutional Review Board (IRB# 00110164), with a waiver for informed consent. The Strengthening the Reporting of Observational studies in Epidemiology (STROBE) guidelines were followed for data reporting [18].

The Sandra Levine Young Women’s Breast Cancer Database at the Levine Cancer Institute (Charlotte, NC, USA) (n = 870) was queried for women aged ≤ 40 years diagnosed between 1 January 2009 and 31 December 2018 with confirmed single, primary stage I–IV breast cancer. Sixty-six deaths were observed, and 56 of these patients had an evaluable CT scan obtained within six months prior to initiation of the first anti-cancer treatment. Because CT-based body composition assessment requires labor-intensive manual segmentation and verification of L3 images, it was not feasible to perform detailed CT-based body composition analysis for all eligible patients in the cohort. Therefore, we employed a nested, matched case–control design to limit the required image review workload. Interested in retaining the observed death events, deceased patients were matched to survivors by age, clinical disease stage, hormone receptor status, and year of diagnosis using an optimal pair-matching algorithm in the ‘optmatch’ software (version 0.10.5) for R version 4.0.2 (R Foundation for Statistical Computing). All matched surviving patients had evaluable pre-treatment CT scans, and a total of 112 radiology scans were reviewed. For this analysis, patients with stage IV disease were excluded to ensure a more homogeneous population and allow clearer evaluation of body composition measures in the context of potentially curable disease. The analytic dataset included 89 individuals (49 survivors, 40 deceased).

### 2.2. Body Composition from Computed Tomography

CT scans were performed as part of clinical care following a standard, single-phase abdomen and pelvis protocol with intravenous contrast in the portal venous phase using a tube voltage of 120 kVp. Body composition measurements were obtained from the CT slice at the level of the third lumbar vertebra (L3), with a slice thickness between 2.5 and 5 mm. The L3 region was chosen due to its established correlation with overall body composition and its reliability in assessing skeletal muscle and adipose tissues [19].

Two trained researchers (RSG, MF) selected the appropriate L3 CT image, which was analyzed using Slice-O-Matic Software version 5.0 (TomoVision^®^, Montreal, QC, Canada). The software used Hounsfield units (HU) to differentiate between muscle and adipose tissue, with manual verification by the researchers. In cases of uncertainty, a radiologist (TS) was consulted for expert opinion. Muscle and adipose tissue areas at L3 were used to estimate the total body tissue volumes, according to established methodology [20].

### 2.3. Definitions and Endpoints

There is no single, universally accepted age cut-off for defining “young women” with breast cancer. In many studies, this group has been operationalized as women diagnosed before age 40 [21,22,23], or age has been used as a surrogate for menopausal status [24]. In the present study, we define “young women” as those aged 40 years or younger at diagnosis, in line with consensus criteria from the European School of Oncology and the European Society for Medical Oncology (ESMO) [25].

The skeletal muscle index (SMI), a measure of muscle quantity, was defined as the total muscle area at L3 (cm^2^) divided by height squared (m^2^). Skeletal muscle density (SMD) was measured as the mean gray-level image (GLI) of the muscle area in HU. The skeletal muscle gauge (SMG) was calculated by multiplying SMI and SMD. Total adipose tissue (TAT) was the sum of the VAT, subcutaneous adipose tissue (SAT), and IMAT measurement. Indexed values were derived by dividing the body composition metric by height squared.

### 2.4. Data Collection

Study data were collected and managed using Research Electronic Data Capture (REDCap, Nashville, TN, USA) electronic data capture tools hosted at Atrium Health. Data were abstracted through electronic medical record (EMR) queries and manual chart review. Manual data abstraction, verification, and recording were performed by a trained study member (MF).

### 2.5. Statistical Analyses

Descriptive statistics included counts and percentages for categorical variables and means (standard deviations) or medians (ranges) for continuous variables. Correlations among body composition metrics were assessed with Pearson correlation coefficients. OS was defined as the time from breast cancer diagnosis to death; surviving individuals were censored at the date of last known contact.

For each body composition metric, hazard ratios and 95% confidence intervals were estimated using Cox proportional hazards models. Because the matching was performed for design considerations rather than the primary statistical analysis, we analyzed the data using standard Cox regression models and adjusted for disease stage and hormone receptor status, rather than using matched-pair (stratified) Cox models. Instead, we fit standard Cox models and adjusted for disease stage and hormone receptor status, selected a priori in consultation with the clinical team, and considered the number of observed events.

We then conducted exploratory threshold analysis for each body composition metric, regardless of statistical significance, when modeled continuously. Optimal cutpoints were identified as those that minimized the *p*-value and maximized the estimated hazard ratios in univariate models. A single cutpoint per metric was retained based on the largest estimated survival difference. No cutpoint was selected when no statistically significant threshold was detected.

Because 5 of the 40 observed deaths were not breast cancer-related, we assessed robustness in a competing risks framework. Breast cancer-specific mortality was modeled as an event of interest, with non-breast-cancer deaths treated as competing events. For each data-derived cutoff from the exploratory threshold analysis, unadjusted sub-distribution hazard ratios were estimated using Fine–Gray models to compare effect sizes with the OS models.

Statistical significance was defined as *p* < 0.05 for all analyses. Given the hypothesis-generating intent of this study, we did not adjust for multiple comparisons. Data analysis was performed using SAS version 9.4 (SAS Institute, Cary, NC, USA).

## 3. Results

### 3.1. Demographic and Clinical Characteristics

Table 1 presents demographic and clinical characteristics of the study cohort (n = 89). Median age at diagnosis was 35 years (IQR, 32–38). Fewer than half (47%) identified as White, and 37% were Black. The majority (78%) were not Hispanic or Latina. Most (65%) were privately insured. At presentation, most had stage II (65%) or stage III (29%) disease, and 30% had triple-negative disease (HER-2-negative, estrogen receptor-negative, progesterone receptor-negative). The majority had undergone surgery (98%), chemotherapy (93%), and radiation therapy (85%). Excluding those with triple-negative disease, 69% (43 of 62) received hormone therapy. Most (67%) were overweight or obese (BMI ≥ 25).

The raw and height-adjusted body composition metrics were highly correlated; strong correlation (ρ > 0.8) was identified between the SM and SMI (ρ = 0.89), IMAT and IMAT index (ρ > 0.99), VAT and VAT index (ρ > 0.99), and SAT and SAT index (ρ = 0.98) (Table 2).

### 3.2. Overall Survival

**Figure 1:**
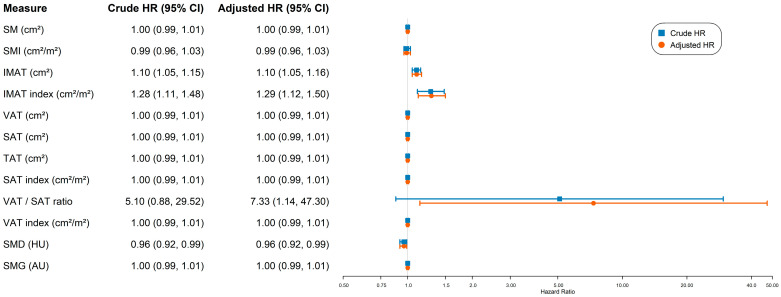
Crude and adjusted hazard ratios for body composition metrics associated with overall survival. Abbreviations: AU: arbitrary unit; HU: Hounsfield units; IMAT: intermuscular adipose tissue; SAT: subcutaneous adipose tissue; SM: skeletal muscle; SMD: skeletal muscle density; SMG: skeletal muscle gauge; SMI: skeletal muscle index; TAT: total adipose tissue; VAT: visceral adipose tissue.

Median follow-up was 8.2 years. In univariate Cox regression models, higher IMAT (HR, 1.10 per cm^2^; 95% CI, 1.05–1.15; *p* < 0.001), higher IMAT index (HR, 1.28 per cm^2^/m^2^; 95% CI, 1.11–1.48; *p* < 0.001), and lower SMD (HR, 0.96 per HU; 95% CI, 0.92–0.99; *p* = 0.02) were significantly associated with worse OS (Figure 1). For example, each 1 cm^2^ increase in IMAT was associated with a 10% (95% CI 5–15%) increase in the hazard of death. After multivariable adjustment, both IMAT (HR, 1.10; 95% CI, 1.05–1.16; *p* < 0.001) and IMAT index (HR, 1.29; 95% CI, 1.12–1.50; *p* < 0.01) remained independently associated with worse OS. The VAT/SAT ratio was also significantly associated with worse OS in the adjusted model (HR, 7.33; 95% CI, 1.41–37.9; *p* = 0.04) (Figure 1). No significant associations were observed for SMI, VAT, SAT, TAT, SMG, or their indices/ratios in either univariate or multivariate models (all *p* > 0.05).

**Figure 2:**
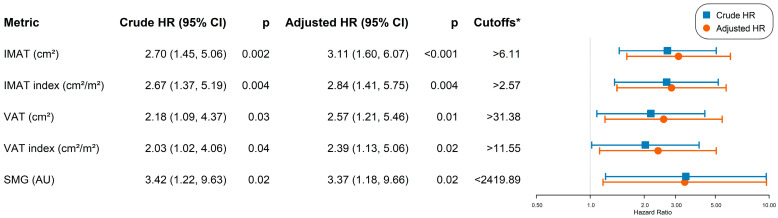
Crude and adjusted hazard ratios for body composition metrics with optimal cutoffs associated with overall survival. Abbreviations: AU, arbitrary unit; HR, hazard ratio; IMAT, intermuscular adipose; SMG, skeletal muscle gauge; VAT, visceral adipose tissue. Note: Adjusted models include adjustments for disease stage and hormone receptor status. * Cutoff values indicate the threshold at which survival estimates are significantly worse.

Threshold analysis identified optimal cutpoints for body composition metrics significantly associated with OS (Figure 2). After adjusting for disease stage and hormone receptor status, high IMAT (>6.11 cm^2^; adjusted HR, 3.11; 95% CI, 1.60–6.07; *p* < 0.001), high IMAT index (>2.57 cm^2^/m^2^; adjusted HR, 2.84; 95% CI, 1.41–5.75; *p* = 0.004), high VAT (>31.38 cm^2^; adjusted HR, 2.57; 95% CI, 1.21–5.46; *p* = 0.014), high VAT index (>11.55 cm^2^/m^2^; adjusted HR, 2.32; 95% CI, 1.14–4.73; *p* = 0.02), and low SMG (<2419.89 AU; adjusted HR, 2.3; 95% CI, 1.13–4.86; *p* = 0.02) were each independently associated with worse OS (Figure 2). No significant cutpoint values were identified for SMI, SAT, TAT, or SMD.

### 3.3. Sensitivity Analysis

Five of the 40 deaths (12.5%) were unrelated to breast cancer. When breast cancer-specific death was considered (treating non-breast-cancer deaths as competing events), all data-derived cutpoints that were significant in the overall survival analyses remained significant, and effect sizes were numerically similar to the OS estimates. The unadjusted sub-distribution hazard ratios (95% CI) were: IMAT (cm^2^), 3.15 (1.63–6.09); IMAT index (cm^2^/m^2^), 2.81 (1.35–5.88); VAT (cm^2^), 2.34 (1.14–4.79); VAT index (cm^2^/m^2^), 2.18 (1.06–4.46); and SMG (AU), 2.75 (1.01–7.55).

## 4. Discussion

This study demonstrated that CT-derived body composition metrics, especially those reflecting muscle quality and adiposity distribution, were significant prognostic markers for overall survival in young women with breast cancer. Specifically, higher IMAT, higher IMAT index, elevated VAT/SAT ratio, and lower SMD were independently associated with poorer survival. In contrast, measures of skeletal muscle mass and total adipose tissue were not predictive of survival in this cohort. Thresholds for IMAT (>6.11), IMAT index (>2.57), VAT (>31.38), VAT index (>11.55), and SMG (<2419.89) were associated with overall survival, providing exploratory cutpoints that may aid hypothesis generation regarding risk stratification in this population. These exploratory findings support integrating quantitative body composition analysis into clinical imaging workflows as practical biomarkers to support individualized cancer care and prognosis. Notably, some metrics, such as VAT and SMG, were not significantly associated with overall survival when modeled as continuous predictors. However, they demonstrated apparent threshold effects only after dichotomization. This pattern underscores the data-driven nature of these cutpoints and the potential for overfitting or spurious findings in a small sample. These exploratory results should therefore be viewed as hypothesis-generating rather than definitive. They require confirmation in larger datasets, ideally using prespecified or externally derived cutpoints. We acknowledge that in this young population with extended follow-up, non-cancer deaths (observed in 12.5% of events) may have contributed to OS associations; the cutoff derived in this exploratory analysis remained statistically significant in a competing risks framework.

To our knowledge, this is the first study to establish cutoff values for CT-derived body composition metrics in young women with breast cancer in the United States. Previous investigations have largely focused on older, postmenopausal, or mixed-age cohorts, highlighting a critical need for age-specific reference data that reflect the distinct metabolic and hormonal context of younger women. Epidemiological differences across age groups highlight the importance of refining risk stratification models tailored to young women with breast cancer.

Among the examined parameters, IMAT demonstrated the strongest associations with mortality. Our findings showed that patients exceeding an IMAT index threshold of >2.57 had a 2.8-fold higher risk of death, which closely parallels Deluche et al.’s report [15] that early breast cancer patients with sarcopenia and a high IMAT index (>3.5) experienced significantly worse prognosis. However, there are notable differences in study populations and methodologies. Our cohort consisted exclusively of young women in the U.S., whereas Deluche et al. [15] studied a broader age range (median age 56) with both pre- and post-menopausal patients from a single European center. Additionally, our analysis derived thresholds specifically for young women, whereas Deluche et al. used pre-specified cutpoints based on their population characteristics.

The prognostic relevance of IMAT and muscle attenuation likely reflects their function as imaging-based indicators of underlying metabolic and inflammatory dysregulation in cancer patients. Increased intermuscular fat infiltration and reduced muscle density have been associated with elevated systemic proinflammatory cytokines (e.g., IL-6, TNF-α), increased oxidative stress, and impaired anabolic signaling, all of which contribute to tumor progression, cachexia, and reduced treatment tolerance [26]. Recent studies confirm that fat infiltration within skeletal muscle serves as a surrogate marker for systemic metabolic dysfunction and subclinical inflammation, predicting worse outcomes across solid tumor populations, including breast cancer [15,27]. Therefore, quantitative body composition metrics provide radiologically quantifiable measures of a patient’s metabolic health and physiological robustness that can inform prognosis and personalize supportive interventions [28].

A higher VAT cut-off value (>31.38) was independently associated with a 2.6-fold increased risk of mortality after adjusting for disease stage and hormone receptor status. To date, prior studies have not reported specific VAT cutoff values for survival among young women with breast cancer, highlighting the novelty of these findings. Although our study identified a statistically significant VAT cutoff, VAT as a continuous measure was not associated with significant differences in OS. This is consistent with prior studies reporting that the adverse impact of visceral adiposity is not consistently observed in early-stage breast cancer [9]. For instance, research suggests that the association between higher VAT and worse survival may be stage-dependent, influencing outcomes more in stage II than stage III disease (age ranging between 27 and 86 years) [16]. These results are in contrast with a prior study in advanced-stage breast cancer, which reported a positive association between visceral adiposity and mortality [29]. VAT exhibits elevated aromatase activity, contributing to local estrogen production that may amplify breast tissue proliferation, particularly in postmenopausal women [30]. Differences in adipose distribution and metabolism by age and menopausal status likely contribute to this variability. Premenopausal women typically store more subcutaneous fat rather than visceral fat, potentially leading to lower systemic inflammation and reduced tumor-promoting effects [31]. Furthermore, limited variability in VAT and a high prevalence of overweight or obese individuals in our cohort may have attenuated our ability to detect VAT-related survival differences [32].

Weinberg et al. proposed SMG as a composite metric that integrates both muscle quantity (mass) and quality (density) derived from CT imaging [33]. While their study found a correlation between SMG and age, its prognostic value for clinical outcomes was not directly evaluated. In contrast, Oliveira Júnior et al. [16] identified SMG as an independent predictor of disease-free survival, supporting its promise as a CT-based biomarker. In our cohort, neither SMG nor SMI was independently associated with OS. However, an SMG threshold of <2419.89 was associated with a higher mortality risk, aligning with earlier observations that lower SMG values confer greater vulnerability. Prior studies have also demonstrated that lower SMG predicts increased treatment-related toxicities, including hematologic and gastrointestinal complications, further reinforcing the clinical value of SMG as a composite index of muscle health [34,35].

SMD is a marker of muscle quality, with prior research correlating myosteatosis, defined by low SMD [36]. We found that low SMD was significantly associated with poorer OS in both univariate and multivariate analyses in our study, and no threshold was identified. This finding is consistent with prior research correlating myosteatosis, defined by low SMD and high IMAT, with worse outcomes in women with breast cancer [33]. In a study after controlling for BMI, age, and comorbid conditions, myosteatosis was associated with a significantly elevated risk of chemotherapy-related toxicities compared to the absence of myosteatosis in women with early breast cancer [37]. Similarly, another study treated SMD as a continuous variable, which revealed lower values in association with the Luminal B and ER−, HER2+ subtypes, implying that improved muscle quality might relate to more favorable disease characteristics [38]. In contrast, another study in non-metastatic breast cancer, including women aged 18–80 years, did not find a relationship of survival with SMD, and the authors implied that muscle fat infiltration is more characteristic of advanced disease and occurs less frequently in early-stage cancer, which may explain its limited prognostic value in non-metastatic patients [14]. Mechanistically, reduced muscle density reflects diminished oxidative capacity, mitochondrial dysfunction, and impaired metabolic flexibility, all linked to systemic inflammation, insulin resistance, and reduced immune competence [39,40]. Additionally, in patients undergoing chemotherapy, myosteatosis has been associated with increased treatment-related toxicity and reduced chemotherapy response. This may suggest that muscle quality is closely linked to both cancer biology and therapeutic efficacy, as patients with lower SMD experience greater metabolic disturbances, higher risk of adverse effects, and reduced response to therapy [14].

Younger age, with protective effects of estrogen on musculoskeletal health and lower prevalence of sarcopenia in our cohort, may explain the lack of significance for SMI. Prior research demonstrating strong associations between low muscle mass and mortality has primarily involved older or postmenopausal women, in whom age-related anabolic resistance and comorbidities amplify the impact of sarcopenia [16]. Younger women may possess greater physiological reserve, mitigating the effects of lower muscle mass on outcomes.

Taken together, our findings support the hypothesis that muscle quality rather than muscle quantity may be more strongly associated with prognosis in young women with breast cancer. The prognostic relevance of IMAT, VAT, SMD, and SMG for poor outcomes supports the emerging view that myosteatosis and reduced muscle attenuation are more sensitive markers of metabolic dysfunction compared to volumetric measures. The mechanistic underpinnings involve the interconnected roles of oxidative capacity, inflammatory signaling, and hormone sensitivity, all of which are reflected in muscle density more accurately than in muscle mass alone [41].

This study is limited by its observational design, which precludes causal inference, and we acknowledge this is an exploratory analysis of twelve metrics, with no adjustments for multiple testing. CT-based assessments capture tissue area and radiodensity but not direct measures of muscle strength or function. As consensus criteria for sarcopenia emphasize muscle strength (e.g., grip strength, gait speed), our inability to assess functional measures restricts comprehensive classification [42]. Combining CT-based measures with objective assessments of muscle strength would provide a more complete picture of body composition and validate imaging biomarkers as functional prognostic tools. Another key limitation is the inability to stratify participants by weight status or SMI, precluding assessment of whether low versus high SMI at diagnosis influences treatment outcomes. We also did not account for post-recurrence anti-cancer therapies, as the cohort comprised patients originally diagnosed with stage I–III disease. The relatively small, single-center, urban cohort may also limit the generalizability of our findings.

We acknowledge that the findings of this study are based on a relatively small sample of 89 patients with 40 OS events. This limited sample size reduces statistical power, increases the risk of overfitting, and constrains the number of covariates that can be reliably included in multivariable models. Our exploratory dichotomization of body composition metrics identified thresholds for some variables (e.g., VAT, SMG) that were not significant when analyzed as continuous predictors, raising the possibility that some observed threshold effects reflect chance findings rather than true biological inflection points. Although matching may have improved comparability between deceased patients and survivors on key prognostic factors, we did not fully leverage the matched design in our survival analyses; we used standard Cox regression adjusted for selected matching variables rather than stratified or pair-clustered Cox models. As a result, the reported confidence intervals may be somewhat optimistic; our variance estimates may underestimate the true level of uncertainty, and the precision and stability of the estimated hazard ratios and cutpoints are limited. In addition, thresholds were not externally or internally validated and this lack of validation increases the risk of overfitting and limits generalizability.

Despite these limitations, routine staging CT scans represent an underused source of prognostic data. Automated extraction of CT body composition metrics at the L3 vertebral level could enable clinicians to identify high-risk individuals with excess adiposity. Future research should prospectively evaluate young breast cancer patients from diagnosis, temporal changes in body composition, and their relationship with outcomes. Multicenter studies with standardized CT acquisition protocols would enhance statistical power and reproducibility.

Recent advances in automated L3 segmentation support the feasibility of incorporating CT-derived body composition into routine oncology workflows. Zhao et al. [43] developed a lightweight algorithm for L3 skeletal muscle segmentation that achieved high agreement with expert-derived standards and substantially reduced processing time compared with deep-learning tools. Although their work focused on rectal cancer and primarily evaluated segmentation performance, it demonstrates that accurate, automated extraction of L3 muscle area is achievable on standard hardware, reducing the manual workload required by Slice-O-Matic-based methods used in our study. Integrating similar automated segmentation approaches with prognostically relevant metrics could enable scalable risk stratification for young women with breast cancer.

## 5. Conclusions

In this first U.S.-based, matched cohort study of young women with non-metastatic breast cancer, higher intramuscular and visceral adiposity and lower skeletal muscle gauge values at diagnosis were independently associated with worse overall survival. Measures of muscle quality and adipose distribution, including IMAT, SMD, and the VAT/SAT ratio, showed the greatest prognostic value, while skeletal muscle mass was less informative. These findings suggest that muscle fat infiltration and visceral adiposity, rather than muscle quantity alone, may be more closely associated with the metabolic and inflammatory milieu that is linked to tumor biology, treatment tolerance, and outcomes.

The identified cutpoints represent an exploratory framework; routine staging CTs, with automated body composition extraction, could potentially enable real-time risk stratification and inform targeted nutritional or exercise interventions. Studies with larger and more diverse cohorts are warranted to validate these cutpoints, confirm their prognostic significance, and determine whether they can be integrated into survivorship care pathways for young women with breast cancer.

## Figures and Tables

**Table 1:** Patient, disease, and treatment characteristics (n = 89).

Age (Years), n (%)	
18–32	25 (28)
33–37	38 (43)
38–40	26 (29)
Race, n (%)	
Black	33 (37)
White	42 (47)
Other	14 (16)
Ethnicity, n (%)	
Hispanic or Latina	16 (18)
Not Hispanic or Latina	69 (78)
Unknown	4 (4)
Primary insurance, n (%)	
Privately insured	58 (65)
Other insurance	31 (35)
Clinical Disease Stage, n (%)	
I	5 (6)
II	58 (65)
III	26 (29)
Hormone Receptor Status, n (%)	
HR+, HER2−	46 (52)
HER2+	13 (15)
HR−, HER2−	27 (30)
Unknown	3 (3)
Treatments: Surgical and Systemic, n (%)	
Surgery	87 (98)
Chemotherapy	83 (93)
Radiotherapy	76 (85)
Hormone therapy *	43 (69)
Immunotherapy	18 (20)
BMI Category (kg/m^2^), n (%)	
Normal weight (18.5–24.9)	29 (33)
Overweight (25–29.9)	24 (27)
Obese (≥30)	36 (40)

Abbreviations: HER2, human epidermal growth factor receptor; HR, hormone receptor. Note: Most numbers and percentages are rounded to the nearest whole number. * Excludes those with triple-negative disease (43 of 62).

**Table 2:** Correlation among body composition metrics.

	SM (cm^2^)	SMI (cm^2^/m^2^)	IMAT (cm^2^)	IMAT Index (cm^2^/m^2^)	VAT (cm^2^)	SAT (cm^2^)	SAT Index (cm^2^/m^2^)	VAT/SAT Ratio	VAT Index (cm^2^/m^2^)	TAT (cm^2^)
SM (cm^2^)	-	0.89	0.07	0.04	0.46	0.51	0.39	0.27	0.44	0.46
SMI (cm^2^/m^2^)		-	0.04	0.05	0.55	0.45	0.50	0.31	0.57	0.51
IMAT (cm^2^)			-	0.99	0.26	0.39	0.36	0.16	0.24	0.39
IMAT index (cm^2^/m^2^)				-	0.27	0.39	0.38	0.17	0.26	0.40
VAT (cm^2^)					-	0.73	0.74	0.73	0.99	0.87
SAT (cm^2^)						-	0.98	0.21	0.71	0.97
SAT index (cm^2^/m^2^)							-	0.23	0.75	0.96
VAT/SAT ratio								-	0.72	0.40
VAT index (cm^2^/m^2^)									-	0.85
TAT (cm^2^)										-

Abbreviations: IMAT, intermuscular adipose tissue; SAT, subcutaneous adipose tissue; SM, skeletal muscle; SMI, skeletal muscle index; TAT, total adipose tissue; VAT, visceral adipose tissue.

## Data Availability

The data that support the findings of this study are available from the corresponding author, [AA], upon reasonable request.

## Abbreviations

The following abbreviations are used in this manuscript:

BMI	Body mass index
CT	Computed tomography
GLI	Gray-level image
HU	Hounsfield units
IMAT	Intermuscular adipose tissue
L3	Third lumbar vertebra
OS	Overall survival
SA	Surface area
SAT	Subcutaneous adipose tissue
SM	Skeletal muscle
SMD	Skeletal muscle density
SMG	Skeletal muscle gauge
SMI	Skeletal muscle index
TAT	Total adipose tissue
VAT	Visceral adipose tissue
